# 

**Published:** 1911-11

**Authors:** 


### CONTENTS.

ORIGINAL COMMUNICATIONS—

Etiology, Pathology and Treatment of Pellagra. By Geo. C. Mizell, M. D. .. 401

The Use of Urotropin in Acute Inflammaions of the Middle Ear. F. Phinizy Calhoun, A. B., M. D., Atlanta, Ga. .................................. 419

Syphilis in Infancy and Early Life. By George K. Varden, M. D., Atlanta, Ga.. 423

Chloroform Death Durigg Child-Birth. By F. G. Hodgson, M. D., Atlanta, Ga. 428

EDITORIALS ................................................ 432

SELECTIONS AND ABSTRACTS ...................................... 435

BOOK REVIEWS ................................................ 456

